# BDNF/TrkB activators in Parkinson's disease: A new therapeutic strategy

**DOI:** 10.1111/jcmm.18368

**Published:** 2024-05-16

**Authors:** Naif H. Ali, Hayder M. Al‐Kuraishy, Ali I. Al‐Gareeb, Athanasios Alexiou, Marios Papadakis, Ali Abdullah AlAseeri, Mubarak Alruwaili, Hebatallah M. Saad, Gaber El‐Saber Batiha

**Affiliations:** ^1^ Department of Internal Medicine, Medical College Najran University Najran Saudi Arabia; ^2^ Department of Clinical Pharmacology and Medicine, College of Medicine Mustansiriyah University Baghdad Iraq; ^3^ Head of Jabir ibn Hayyan Medical University Najaf Iraq; ^4^ University Centre for Research and Development, Chandigarh University Mohali Punjab India; ^5^ Department of Research and Development Funogen Athens Greece; ^6^ Department of Research and Development AFNP Med Wien Austria; ^7^ Department of Science and Engineering Novel Global Community Educational Foundation Hebersham New South Wales Australia; ^8^ Department of Surgery II University Hospital Witten‐Herdecke, University of Witten‐Herdecke Wuppertal Germany; ^9^ Department of Internal Medicine College of Medicine, Prince Sattam bin Abdulaziz University Al‐Kharj Saudi Arabia; ^10^ Department of Internal Medicine, College of Medicine Jouf University Sakaka Saudi Arabia; ^11^ Department of Pathology, Faculty of Veterinary Medicine Matrouh University Matrouh Egypt; ^12^ Department of Pharmacology and Therapeutics, Faculty of Veterinary Medicine Damanhour University Damanhour Egypt

**Keywords:** BDNF, BDNF/TrkB activators, Parkinson's disease, TrkB

## Abstract

Parkinson's disease (PD) is a neurodegenerative disorder of the brain and is manifested by motor and non‐motor symptoms because of degenerative changes in dopaminergic neurons of the substantia nigra. PD neuropathology is associated with mitochondrial dysfunction, oxidative damage and apoptosis. Thus, the modulation of mitochondrial dysfunction, oxidative damage and apoptosis by growth factors could be a novel boulevard in the management of PD. Brain‐derived neurotrophic factor (BDNF) and its receptor tropomyosin receptor kinase type B (TrkB) are chiefly involved in PD neuropathology. BDNF promotes the survival of dopaminergic neurons in the substantia nigra and enhances the functional activity of striatal neurons. Deficiency of the TrkB receptor triggers degeneration of dopaminergic neurons and accumulation of α‐Syn in the substantia nigra. As well, BDNF/TrkB signalling is reduced in the early phase of PD neuropathology. Targeting of BDNF/TrkB signalling by specific activators may attenuate PD neuropathology. Thus, this review aimed to discuss the potential role of BDNF/TrkB activators against PD. In conclusion, BDNF/TrkB signalling is decreased in PD and linked with disease severity and long‐term complications. Activation of BDNF/TrkB by specific activators may attenuate PD neuropathology.

## Graphical abstract

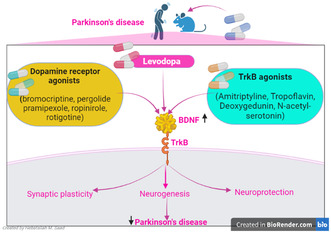

## 1 INTRODUCTION

Since James Parkinson initially described the phenomenon of shaking palsy more than 200 years ago, different research in this field has been done to identify the pathologic mechanism of this disease.^1^, ^2^ Later on, shaking palsy was renamed as Parkinson's disease (PD).^3^ Pathognomonic features of PD were written by medical treatise many centuries ago in the Bible, Egyptian papyrus and Galen writing, they discover symptoms similar to those of PD.^4^ Since 1450 years ago, Imam Ali said that ‘Life is limited and no one will exceed what is destined for him, so hurry before the deadline runs out’ proposed that ageing is linked with cognitive impairment. In the 17th and early 18th centuries, many researchers including Franciscus Sylivus, John Hunter and Hieronymus Gaubius wrote about some elements of PD.^4^ In 1817, James Parkinson reported six patients with paralysis agitans.^4^ Between 1868 and 1881, many neurologists including Jean‐Martin Charcot, Kinnier Wilson and Trousseau gave more clinical insight into PD. In 1912, Lewy bodies a hallmark of PD were primarily described by Frederic Lewy.^5^ Konstantin Tretiakoff in 1919 reported that degeneration of substantia nigra was associated with PD neuropathology.^5^ Biochemical analysis of dopaminergic neurotransmission was extensively studied by Arvid Carlsson and Hornykiewicz in 1950.^6^ Remarkably, in 1997 Trojanowski and Spiillantini recognized that alpha‐synuclein (α‐Syn) is the main component of Lewy bodies and is involved in the pathogenesis of PD.^7^ The treatment strategy for PD was surgery and the use of anticholinergic drugs till the discovery of levodopa by Casimir in 1911 but was not used in clinical practice till 1967.^8^ In recent times, PD has been regarded as a multi‐etiological pathological condition with tentative aetiopathogenesis due to the detection of different molecules involved in PD neuropathology. PD neuropathology is complex and may be started outside the central nervous system (CNS).^2^, ^9^, ^10^ Till a recent time, only symptomatic treatments are available in the management of PD patients. Targeting of growth factors such as brain‐derived neurotrophic factor (BDNF) and its receptor tropomyosin receptor kinase type B (TrkB) by specific activators such as dopamine receptor agonists may attenuate PD neuropathology.^11^ Therefore, aim of this perspective was to revise the potential role of BDNF/TrkB activators against PD.

## 2 PATHOGENESIS AND CLINICAL FEATURE OF PD

PD is characterized as a long‐term and advancing neurodegenerative disorder affecting the brain.^12^ PD is distinguished by motor manifestations such as tremors, rigidity, postural instability and bradykinesia.^13^ In PD, non‐motor symptoms such as dementia, cognitive dysfunction, sleep disorders and depression may emerge several decades before the advancement of motor symptoms.^14^, ^15^ Among people over 60, PD affects 1%–3% of the population.^16^ The primary risk factor for PD and a strong predictor of the disease's severity is ageing.^17^ Early onset PD refers to the development of PD before the age of 50, while the term juvenile PD is used when PD develops before the age of 21.^18^ Notably, PD is observed to be higher in men relative to women primarily because of the elevated levels of oestrogen in women, which plays a significant role in providing neuroprotection against the neuropathology associated with PD.^19^ Furthermore, the frequency of Parkinson's disease is higher in Western countries in comparison with the Asian population.^20^

The neuropathology of Parkinson's disease (PD) arises from the gradual deterioration of dopaminergic neurons located in the substantia nigra, as well as the build‐up of Lewy bodies within the survival motor neurons.^21^ The primary cause of Lewy bodies is the deposition of α‐Syn, which is also present in other neurological conditions known as synucleinopathies.^22^ Notably, substantia nigra motor dopaminergic neurons are lost by 70% before Parkinson's disease symptoms appear.

**FIGURE 1:**
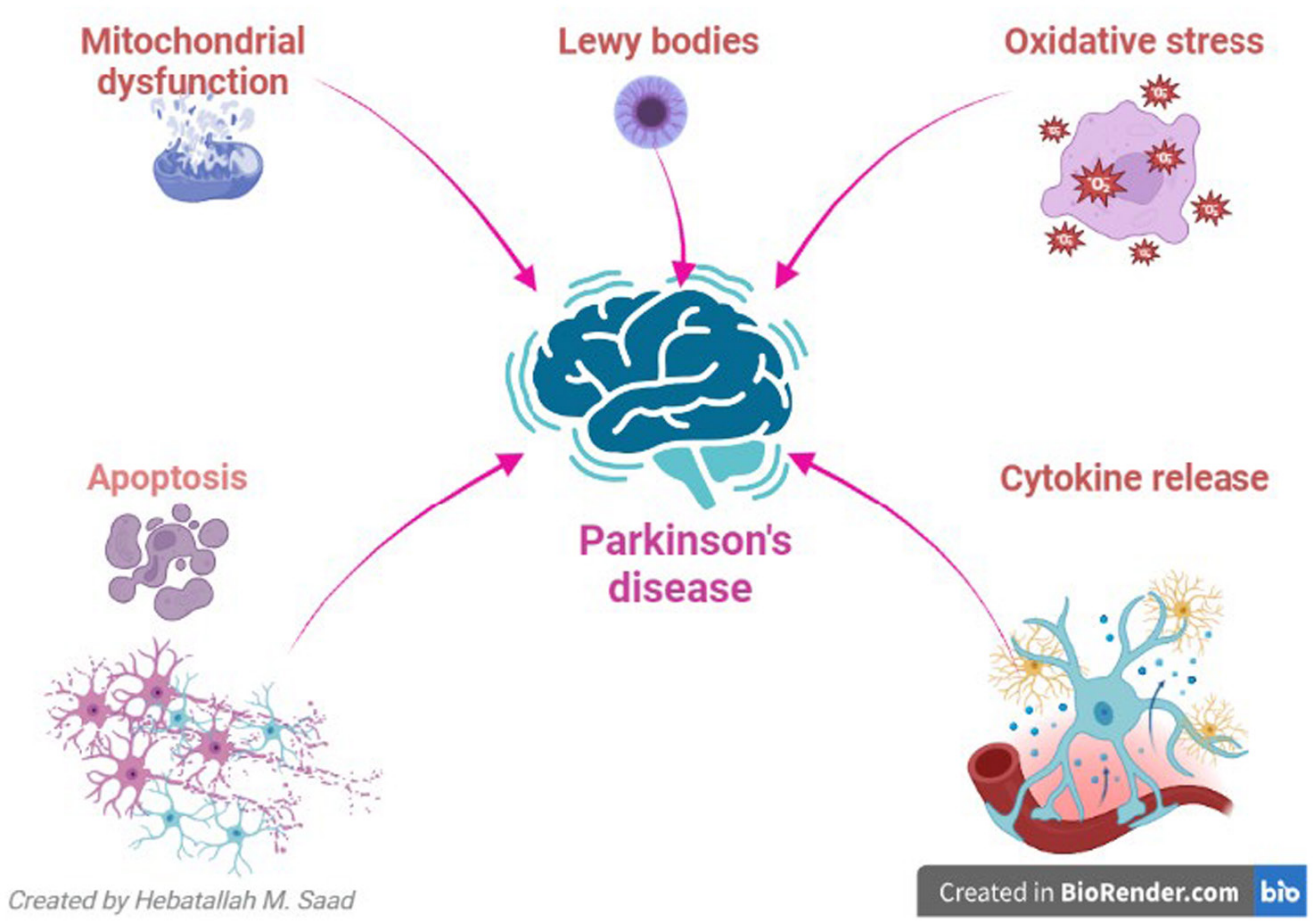
Pathophysiology of Parkinson's disease (PD): Accumulation of Lewy bodies and associated oxidative stress, mitochondrial dysfunction, apoptosis and cytokines release are involved in the pathogenesis of PD.

The role and impact of α‐Syn in PD is a subject of debate, with the possibility of it either being pathogenic or compensatory, leading to an increase to mitigate the loss of dopaminergic neurons.^23^ Idiopathic (sporadic) PD, which constitutes 90% of cases, and familial PD, which accounts for only 10% of cases, are the two distinct types of PD that have been identified. Mutation of α‐Syn is related to the progression of familial PD.^24^ It has been stated that genetic changes linked to PD are present from early embryonic life and predispose individuals over the age of 60 to develop PD.^25^ Genetic modification may interact with various environmental factors in the pathophysiology of PD.^25^ As a result of these genetic discoveries, PD was reclassified as a heterogeneous neurological disease.^26^ Three temporal phases have been proposed to be involved in the pathogenesis of PD: triggers (e.g., environmental toxins), facilitators (e.g., peripheral inflammation) and aggravators (e.g., autophagy dysfunction).^27^ For instance, changes in the nasal microbiome and dysbiosis of the gut encourage the deposition of α‐Syn and the emergence of PD's non‐motor symptoms.^28^ The chronic metabolic disease‐related inflammatory process promotes neuroinflammation, degeneration of dopaminergic neurons in the substantia nigra and α‐Syn accumulation.^29^ Furthermore, PD neuropathology may be promoted by impaired autophagy, which lowers α‐Syn clearance.^30^ Moreover, oxidative stress, apoptosis, mitochondrial dysfunction and growth factor dysfunction all play a role in the pathophysiology of PD.^31^ Thus, growth factors' control over mitochondrial dysfunction, oxidative stress and apoptosis may offer a novel approach to the treatment of PD (Figure 1).

## 3 BDNF OVERVIEW

**FIGURE 2:**
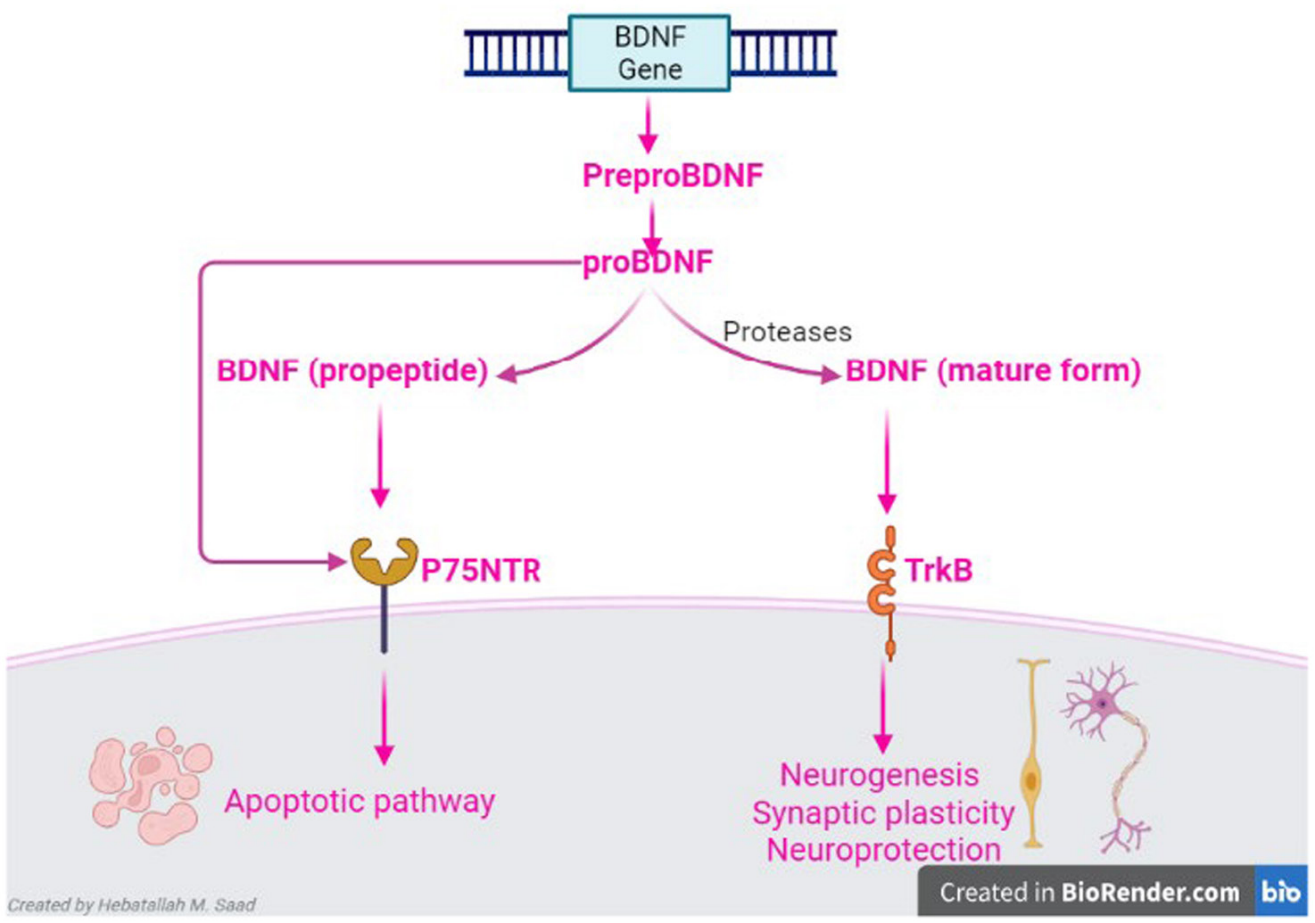
Differential effect of BDNF and pro‐BNDF: *The BNDF* gene promotes the expression of preproBDNF mRNA and increases the synthesis of proBDNF, which is catalysed to form mature BDNF and propeptide BDNF. Mature BDNF activates tyrosine kinase receptor B (TrkB) leading to the neuroprotective effect through induction of neurogenesis and synaptic plasticity. However, propeptide BDNF (proBDNF) induces apoptosis through the activation of the p75 neurotrophin receptor (p75NTR).

BDNF is a member of the neurotrophins protein family that is involved in neuronal regulation and memory performance.^32^ BDNF acts on tyrosine kinase receptor B (TrkB) and p75 neurotrophin receptor (p75NTR).^32^ BDNF is released from peripheral tissues and the CNS.^33^ The interaction between BDNF and TrkB improves neurogenesis, synaptic plasticity and neuroprotection and could be a therapeutic target against mental and neurodegenerative diseases.^34^ BDNF is synthesized from pro‐BNDF by proteolytic cleavage including plasmin.^17^ Pro‐BNDF via activation of p75NTR triggers apoptotic pathway in glia and peripheral neurons^34^ (Figure 2). The regulation of glucose homeostasis and energy balance is closely linked to the peripheral action of BDNF.^35^ BDNF regulates mitochondrial function and thermogenesis; therefore, it mediates the effects of muscle exercise on the cognitive function.^36^ Noteworthy, ageing, chronic stress and neurodegenerative diseases inhibit the synthesis of BDNF.^37^ However, exercise and antidepressant agents improve the synthesis of BDNF.^38^

Peripheral BDNF is derived from platelets, vascular and epithelial cells, leukocytes and macrophages.^39^ Peripheral BDNF cannot cross BBB and less contributes to brain regulation.^40^ However, derangement of BBB integrity in neurodegenerative diseases facilitates the transport of BDNF across the BBB into the brain.^41^ Consequently, peripheral and central BDNF are related, and mental disorders may be replicated by serum BDNF. In this concept, several investigations have shown that a wide range of neuropsychiatric disorders are impacted by serum BDNF.^42^ Moreover, neuropsychological and neuroimaging investigations showed that in healthy subjects, there is an association between central and peripheral BDNF.^39^ Furthermore, many drugs influence the levels of BDNF in the serum and its expression in various neurological diseases. In schizophrenia patients, sedative and hypnotic benzodiazepines like lorazepam lower serum levels of BDNF.^43^ But mood‐stabilizing medications like lithium boost BDNF expression.^44^ However, there is conflicting evidence regarding whether or not these medications influence peripheral or central BDNF, as well as the duration of time required for this effect to manifest.

## 4 BDNF AND NEURODEGENERATIVE DISEASES

It has been shown that BDNF is intricate in many neurological disorders such as Alzheimer's disease (AD), multiple sclerosis (MS) and depression.^45^ Notably, many neurodegenerative diseases impair the production of BDNF in the brain; in turn, downregulation of BDNF exaggerates the neurodegenerative process.^45^

### 4.1 Role of BDNF in AD

Alzheimer's disease is the most prevalent neurodegenerative disease characterized by cognitive impairment and memory dysfunction.^46^, ^47^, ^48^ AD neuropathology is characterized by extracellular accumulation of mutant amyloid beta (Aβ) peptide and intracellular accumulation of hyper‐phosphorylated tau protein which form neurofibrillary tangles (NFTs).^22^, ^49^, ^50^, ^51^ Of note, synaptic abnormalities are involved in AD neuropathology and linked with disease severity and progression of Aβ and NFTs. Both Aβ and NFTs alter the synapses and neuronal circuit resulting in cognitive dysfunction and impairment of the synthesis and the release of BDNF from brain neurons.^52^, ^53^, ^54^ In addition, depletion of neuronal BDNF promotes the accumulation of Aβ and NFTs and induces the development of synaptic dysfunction, apoptosis and neuroinflammation.^55^ Deficiency of BDNF synthesis is extremely reduced in AD disease due to the progressive intracellular accumulation of NFTs and associated neurodegeneration.^56^ Moreover, a postmortem study showed that BDNF mRNA levels are reduced in the entorhinal cortex and parietal cortex of AD patients.^57^ Of interest, low BDNF mRNA levels in the temporal cortex, hippocampus and CSF are correlated with degeneration of the basal forebrain cholinergic neurons in AD models.^58^ Reduction of brain proBDNF is also correlated with the progression of AD neuropathology.^59^ A case–control study included patients with different neurological disorders such as AD, Lewy body dementia, vascular dementia and frontotemporal dementia showed that BDNF serum levels were deregulated in those patients compared to healthy controls.^60^ Subsidiary to this concept, diverse studies specified that BDNF serum level is functionally altered in different neurodegenerative diseases.^61^ It has been stated that the reduction of BDNF signalling through TrkB impairs spatial memory and long‐term potentiation (LTP).^62^ However, BDNF signalling through p75NTR accelerates long‐term depression (LTD) in AD.^63^ Therefore, the effect of BDNF on the cognitive function in AD is differential according to the activated receptors. Interestingly, BDNF mainly acts on TrkB whereas proBDNF mainly activates p75NTR leading to an opposite effect.^64^

These findings indicated that BDNF signalling is reduced in AD due to the progressive neurodegeneration, and implicated in the development of cognitive impairment.

### 4.2 Role of BDNF in MS

Multiple sclerosis is autoimmune‐mediated demyelination of white matter of the brain and spinal cord due to the development of auto‐reactive T and B cells.^65^ Immune dysregulation induces progressive injury of oligodendrocytes of the neurons leading to demyelination and neurodegeneration.^66^, ^67^, ^68^ BDNF has been implicated in the modulation of neuroinflammation and protection of oligodendrocytes in MS neuropathology.^69^ A preclinical study demonstrated that BDNF knockout mice experience deficits in oligodendrocyte proliferation and the production of myelin protein.^70^ Additionally, the expression of BDNF mRNA is higher near the active MS lesions than in the same inactive lesions.^71^ These results indicated that the expression of BDNF in MS may increase to counterbalance the neuroinflammation and associated neurodegeneration. It has been observed that BDNF serum levels were raised in MS individuals during relapse phase.^72^ However, in progressive MS, BDNF serum levels are reduced in MS patients.^73^ Furthermore, CSF BDNF levels are extremely decreased in MS individuals relative to healthy controls.^74^ Conversely, other studies indicated that CSF BDNF levels are upregulated in MS individuals relative to healthy controls.^75^ Therefore, there is a strong controversy regarding the potential role of BDNF in MS that requires further studies.

Moreover, alteration of BDNF signalling has been shown in other neurodegenerative diseases including amyotrophic lateral sclerosis (ALS) and Huntington's disease (HD). Mounting evidence showed that BDNF has a protective effect on motor neurons, though upregulation of BDNF signalling may have detrimental effects on the motor neurons by enhancing glutamate neurotoxicity. Inhibition of BDNF signalling can protect motor neurons in early ALS. However, activation of BDNF signalling in late ALS attenuates deaths of motor neurons.^76^, ^77^, ^78^ It has been indicated that BDNF signalling is highly decreased in individuals with HD, and low BDNF predicts the incidence of HD.^79^ However, a case–control study illustrated that CSF BDNF level was not differed in HD individuals in comparison with controls.^80^ BDNF dysregulation has been shown to be present in neurodegenerative disorders, thus indicating that plasma/CSF BDNF levels might serve as indicators of the severity and progression of these disease.

**FIGURE 3:**
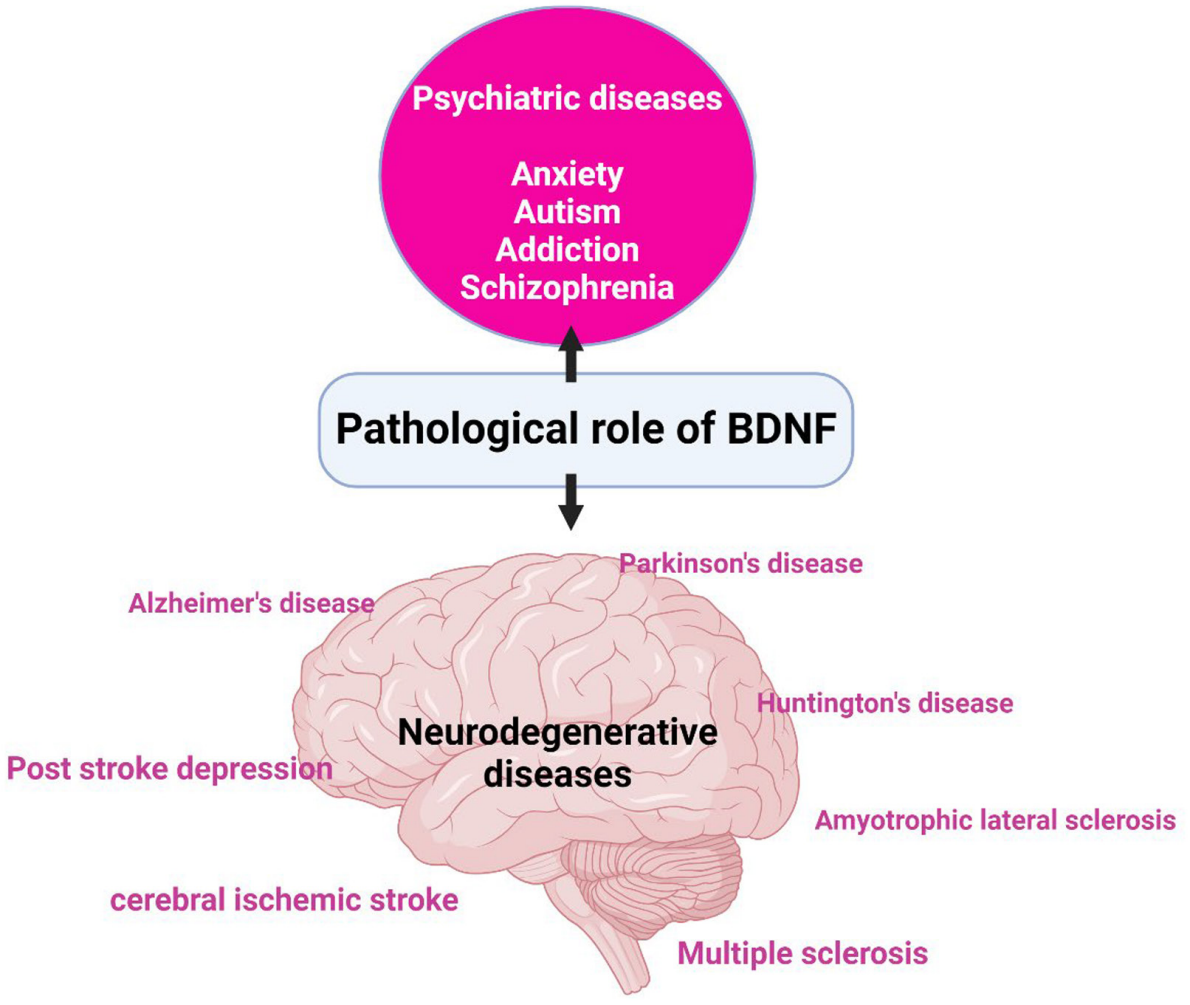
Role of BDNF in different neurological disorders.

Thus, BDNF is pathological in many psychiatric, neurodegenerative and other neurological diseases (Figure 3).

## 5 BDNF IN PD

BDNF through non‐canonical signalling can induce an acute effect by modulating the neurotransmission and synaptic plasticity though; the chronic effect of BDNF through canonical signalling increases different gene expression.^80^ BDNF‐signalling pathways are highly reduced in ageing and predispose to the progression of neurodegenerative diseases as PD.^81^ BDNF promotes the survival of the dopaminergic neurons in the substantia nigra and enhances the functional activity of striatal medium spiny neurons which are the principal afferent neurons from the substantia nigra.^82^ It has been illustrated that BDNF can improve the production and uptake of dopamine.^82^ Preclinical findings support that BDNF improves the viability of dopaminergic neurons in the substantia nigra.^83^ Thus, in the substantia nigra, BDNF signalling has a neuroprotective impact that prevents dopaminergic neurons from degeneration. It has been reported that deficiency of the TrkB receptor triggers the degeneration of dopaminergic neurons in the substantia nigra and accumulation of α‐Syn in mice.^84^ Preclinical findings illustrated that long‐term vibration training improves motor function in the 1‐methyl‐4‐phenyl‐1,2,3,6‐tetrahydropyridine (MPTP) mouse model by increasing BDNF expression.^85^ In addition, cysteamine has a neuroprotective against the MPTP mouse model by increasing BDNF expression and dopamine hydroxylase.^86^ Cao et al.^87^ stated that activation of the transcription factor NF‐E2‐related factor‐2 (Nrf2) and inhibition of the DNA methyl‐binding protein 2 (MeCP2) have been observed to exert regulatory control over the upregulation of BDNF. The transcription of BDNF, which is regulated by Nrf2/MeCP2, is responsible for reversing the reduced levels of BDNF expression in both MPTP‐treated SH‐SY5Y cells and MPTP‐intoxicated mice. Administration of the Nrf2 activator sulforaphane diminishes dopaminergic neurotoxicity in mice treated with MPTP by stimulating BDNF and repressing MeCP2 expression. Furthermore, in MPTP‐treated mice, silencing of MeCP2 expression reduces dopaminergic neurotoxicity by promoting the expression of BDNF and Nrf2. Therefore, a novel therapeutic strategy for Parkinson's disease (PD) might involve suppressing MeCP2 and/or activating BDNF transcription by Nrf2 activators.^87^

In clinical settings, postmortem analysis revealed that mRNA of BDNF was highly reduced in the substantia nigra of PD brains.^11^ The BDNF serum level was significantly lower in the early stages of PD patients compared to controls in a case–control study involving 47 PD individuals and 23 healthy controls.^88^ Later, as PD neuropathology developed, the serum level of BDNF rose and was linked to the severity of the illness.^88^ Increasing BDNF serum level in the late stage of PD could be a compensatory mechanism to reduce the degeneration of dopaminergic neurons in the substantia nigra and associated neuroinflammation. Furthermore, PD onset and progression are associated with a decrease in peripheral BDNF/TrkB pathway.^89^ Expression of BDNF and TrkB in the peripheral blood lymphocytes was highly reduced in 28 PD individuals compared to 28 healthy controls.^89^ However, chronic treatment with L‐dopa in PD patients enhances the expression of BDNF and TrkB.^89^ Interestingly, a case–control study involved 48 PD patients and 24 healthy control subjects disclosed that CSF BDNF level was increased in PD patients compared to control.^90^ Furthermore, a case–control study on 97 PD individuals and 102 controls indicated that BDNF serum level is declined in PD individuals and associated with cognitive dysfunction compared to controls.^91^ Therefore, augmentation of BDNF/TrkB signalling in the late stage of PD might be due to the treatment with L‐dopa. Furthermore, BDNF is correlated with specific presentations in PD patients like restless leg syndrome and depression.^92^, ^93^ A cross‐sectional study included 53 PD patients with restless leg syndrome, 196 PD patients without restless leg syndrome and 326 matched controls showed that BDNF serum level was reduced in PD patients with restless leg syndrome.^92^ A cohort study on PD patients illustrated that BDNF serum level was reduced mainly in PD women patients with depression and motor dysfunctions.^93^ Thus, serum level of BDNF could be a useful marker for the detection of the development of depression in PD patients. An association between a lower level of peripheral BDNF and the incidence of PD has been supported by a systematic review, which shows that blood levels of BDNF are decreased in patients with PD despite a variety of influencing factors.^94^ Miller et al.^11^ observed that higher expression of α‐Syn in postmortem PD patients was associated with a considerable decline in BDNF expression. Of note, α‐Syn downregulates BDNF expression and its downstream signalling.^95^ Retrograde transport of BDNF and TrkB are reduced in neurons overexpressing α‐Syn.^95^ Hence, improvement of BDNF/TrkB signalling can reduce α‐Syn neuropathology. In addition, inhibition of α‐Syn by fingolimod improves BDNF expression in mutant mouse model.^96^, ^97^ As well, exercise‐induced BDNF expression mitigates the aggregation of α‐Syn in animal model study.^98^ Deep brain stimulation promotes the release of BDNF despite the presence of α‐Syn.^99^

Furthermore, in the earliest phases of PD, proBDNF may serve as a diagnostic marker. A longitudinal study follow‐up of 165 patients with tremors and/or bradykinesia for 1 year indicated that proBDNF serum level was increased in individuals developing PD in comparison with other patients. However, BDNF was not significantly altered in patients developing PD compared to other patients.^100^ Therefore, the estimation of the levels of both proBDNF and BDNF are more important diagnostic values than BDNF when it is evaluated alone. In PD and other neurodegenerative diseases, the conversion of proBDNF to BDNF is inhibited mainly in the striatum and hippocampus leading to an imbalance of proBDNF BDNF expression.^101^ A preclinical study found that the expression of proBDNF mRNA is augmented in rotenone‐induced striatal injury in rats.^102^ Of interest, intracellular furin/proprotein convertases and extracellular proteases like plasmin and matrix metallopeptidases proteolytically cleave proBDNF into BDNF. However, accumulated α‐Syn inhibits plasmin by upregulating of plasminogen activator inhibitor‐1.^103^ Therefore, proBDNF is augmented in PD leading to neuronal apoptosis in the substantia nigra.

Moreover, there is a tight connection between the brain and gastrointestinal tract through the gut–brain axis. It has been illustrated that α‐Syn accumulation is initially started in the enteric nervous system and can transported retrogradly via vagus nerve to the brain. In addition, gut dysbiosis may induce systemic inflammation and the development of neuroinflammation in PD.^104^, ^105^ Of note, peripheral BDNF signalling regulates neural survival and gut function. However, accumulated α‐Syn in the enteric nervous system inhibits the expression of BDNF in the gut. Supporting this claim, animal and human studies showed that BDNF signalling is reduced in the gut and correlated with gastrointestinal disorders in PD by inducing gut inflammation and dysregulation of gut microbiota.^106^ Bistoletti et al.^107^ confirmed that antibiotic‐induced gut dysbiosis inhibits the expression of BDNF in both the gut and brain in mice. Therefore, alteration of the gut–brain axis adversely affects the pathogenesis of PD by reducing the expression of BDNF.

These findings demonstrated that PD patients have severe dysregulation of BDNF signalling (Table 1).

**TABLE 1:** Dysregulation of proBDNF/BDNF signalling in PD.

Ref.	Type of study	Findings
[82, 83]	Preclinical	BDNF improves the release and the viability of dopaminergic neurons in the substantia nigra
[85]	Preclinical	Long‐term vibration training improves motor function in the MPTP PD mouse model by increasing BDNF expression
[86]	Preclinical	Cysteamine has a neuroprotective against the MPTP mouse model by increasing BDNF expression
[87]	Preclinical	Nrf2 activator sulforaphane reduces dopaminergic neurotoxicity in MPTP‐treated mice via activation of BDNF
[102]	Preclinical	The expression of proBDNF mRNA is augmented in rotenone‐induced striatal injury in rats
[107]	Preclinical	Antibiotic‐induced gut dysbiosis inhibits the expression of BDNF in both the gut and brain
[11]	Clinical	BDNF is highly decreased in the substantia nigra of PD brains
[88, 89, 91]	Clinical	In early stage PD patients, the serum level of BDNF is significantly lower than in control individuals
[90]	Clinical	CSF BDNF level is raised in PD patients compared to control
[93]	Clinical	BDNF serum level is decreased in PD patients with depression
[100]	Clinical	proBDNF serum level is increased in PD individuals
[106]	Clinical	BDNF signalling is reduced in the gut and correlated with gastrointestinal disorders in PD individuals

## 6 THE NEUROPROTECTIVE MECHANISMS OF BDNF IN PD

**FIGURE 4:**
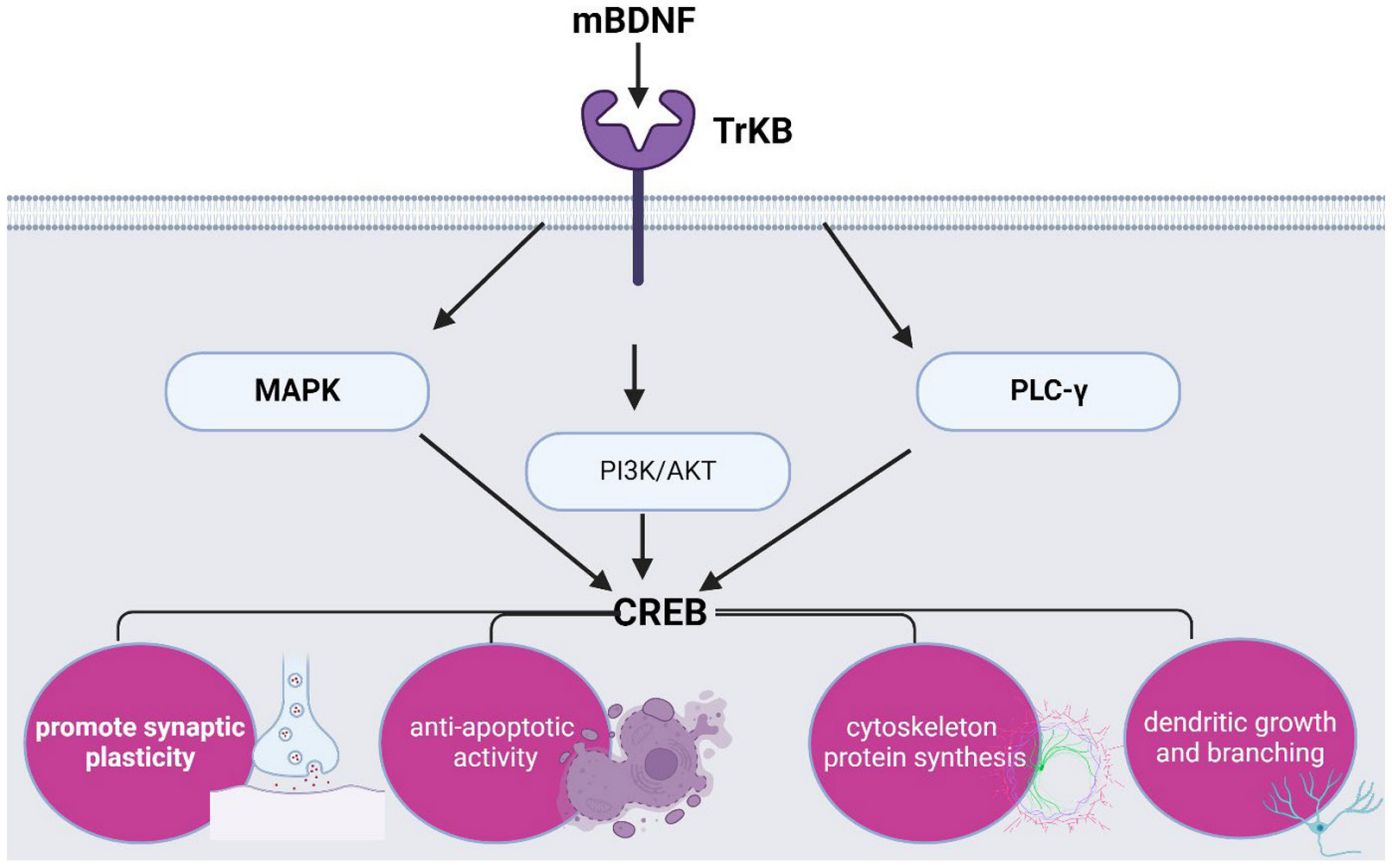
BDNF signalling: BDNF through activation of the TrkB receptor induces the expression of various signalling downstream including mitogen‐activated protein kinase (MAPK), phosphatidylinositol 3 kinase (PI3K) and phospholipase C‐γ (PLC‐γ). These signalling activate the expression of cAMP‐responsive element binding protein (CREB) which promotes synaptic plasticity, anti‐apoptotic activity, enhancement of cytoskeleton protein synthesis and control of dendritic growth and branching.

It has been shown that BDNF via activation of the TrkB receptor induces the expression of various signalling downstream including mitogen‐activated protein kinase (MAPK), phosphatidylinositol 3 kinase (PI3K) and phospholipase C‐γ (PLC‐γ).^108^ These signalling activate the expression of cAMP‐responsive element binding protein (CREB) which promote synaptic plasticity, anti‐apoptotic activity, enhancement of cytoskeleton protein synthesis, and control dendritic growth and branching^108^ (Figure 4).

BDNF promotes the survival of dopaminergic neurons in the substantia nigra by inhibiting apoptosis of the nigrostrial pathway.^109^ In addition, BDNF promotes the expression of D3 receptor and tyrosine hydroxylase.^110^ Therefore, a reduction in the expression of BDNF is linked with degeneration of dopaminergic neurons in the substantia nigra. Of interest, polymorphism of BDNF is linked to higher incidence of familial PD.^81^

Concerning the molecular role of BDNF on PD neuropathology, BDNF and associated downstream affect the pathogenesis of PD. CREB is a cellular transcription factor that binds DNA cAMP response element affecting gene expression. CREB mediates dopamine‐induced neuroplasticity.^111^ Xu et al.^112^ found that inactivation of CREB in the dopaminergic neurons of the substantia nigra was higher in postmortem PD patients. Consistent with these findings, Zhong et al.^113^ illustrated that CREB activator roflupam attenuates the degeneration of the dopaminergic neurons in the substantia nigra in PD model. Besides, the PI3K which is involved in the transport of phosphatidylinositol and other phospholipid from endoplasmic reticulum to other membranes is reduced in PD. In the MPTP PD model, PI3K is downregulated due to oxidative stress.^114^ Notably, the reduction of PI3K by ageing predisposes to the development of PD.^114^ Also, downregulation and expression of PLC‐γ are common in PD due to the neurotoxic effects of α‐Syn.^115^ Hence, restoration of CREB and its downstream by BDNF can prevent the development and progression of PD.

Taken together, BDNF has a vital neuroprotective role in preventing PD. Therefore, augmentation of BDNF signalling by activators could be a novel therapeutic strategy in the treatment of PD.

## 7 ACTIVATORS OF BDNF/TrkB SIGNALLING IN PD

Activation of deficient BDNF/TrkB signalling with specific agonists and small molecules displays promising treatment effects. Thus, searching for BDNF/TrkB agonists is required to mitigate PD pharmacotherapy.

### 7.1 Levodopa

Levodopa (L‐dopa) is one of the most common drugs used in the management of PD by increasing the synthesis of dopamine in the substantia nigra. L‐dopa is used in combination with peripheral decarboxylase inhibitors to promote the delivery of L‐dopa into the CNS and reduced peripheral conversion of L‐dopa to dopamine.^116^ It has been reported that chronic treatment with L‐dopa increases the expression of BDNF.^117^ L‐dopa‐induced dyskinesia may be due to the overexpression of BDNF.^117^ However, a preclinical study confirmed there is no significant correlation between dyskinesia and BDNF level.^118^ Conversely, the administration of L‐dopa in the MPTP monkey model was associated with a reduction of BDNF expression independent of dyskinesia.^118^ A previous preclinical study revealed that L‐dopa increases the expression of BDNF within 2 h that was sustained for 16 h in the striatum in a mouse model.^119^ Thus, increasing the synthesis of dopamine in the dopaminergic neurons by L‐dopa may enhance the expression of BDNF in PD. Similarly, L‐dopa attenuates 6‐OHDA‐induced PD model in rats by increasing the expression of BDNF in the striatum.^120^ Repeated administration of L‐dopa increases BDNF expression in the striatum.^120^ This finding may explain the neuroprotective effect of L‐dopa through the enhancement of synaptic plasticity in PD. BDNF can induce the expression of the D3 receptor in the striatum of PD rat model.^121^ As well, BDNF enhances dopaminergic neurotransmission in other brain regions including the limbic system.^122^ Narita et al.^123^ illustrated that methamphetamine‐induced behavioural changes are mediated by increasing the expression of BDNF. Therefore, induction of BDNF by L‐dopa may aggravate neuropsychiatric disorders in PD.

### 7.2 Monoamine oxidase type B (MAO‐B) inhibitors

Monoamine oxidase type B inhibitors (rasagiline, selegiline and safinamide) are a class of medications used in the management of PD, depression, anxiety disorders and panic disorder by MAO which is responsible for the metabolism of dopamine. MAO‐B inhibitors are more selective and do not cause hypertensive crises such as non‐selective MAO inhibitors.^124^ Selegiline was approved for the management of PD in 2006, it is a selective MAO‐B inhibitor, though selegiline dose more than 20 mg/day can also inhibit MAO‐A. Therefore, a large dose of selegiline leads to a loss of its selectivity.^125^ Selegiline has neuroprotective effects by inhibiting MAO‐B expressed in glial cells.^125^ Preclinical studies confirmed that selegiline has a neuroprotective effect on dopaminergic neurons by activating the release of BDNF.^124^, ^125^ Selegiline enhances BDNF signalling in cultured mouse astrocytes independent of MAO‐B inhibition.^124^ As well, selegiline has a trophic effect on the dopaminergic neurons by increasing the expression of BDNF in dopaminergic neurons.^125^ Selegiline attenuates motor deficit in mice with the MPTP PD model by increasing the expression of BDNF and activating TrkB in the motor neurons of substantia nigra.^126^ Indeed, selegiline through activation of TrkB activates antioxidant signalling in SH‐SY5Y cell lines.^127^

Rasagiline is an irreversible MAO‐B inhibitor that can be used as a monotherapy in the management of early and advanced PD. Unlike selegiline, rasagiline is not metabolized to levomethamphetamine which induces amphetamine‐induced hallucination.^128^ Rasagiline preferentially activates the expression and the release of BDNF in animal and human studies.^129^ It has been reported that rasagiline increases CSF BDNF in PD patients.^125^ Rasagiline attenuates the interaction between α‐Syn and BDNF/TrkB with subsequent increasing the expression of BDNF.^130^ Markedly, α‐Syn blocks TrkB and prevents the biological neuroprotective effect of BDNF on the dopaminergic neurons in the substantia nigra.^130^ Suppression of MAO‐B by rasagiline reduces the formation of dopaminergic toxic metabolites which are involved in the degeneration of dopaminergic neurons in the substantia nigra.^130^ Rasagiline rescues neurons by activating BDNF and other neurotrophic factors.^131^ In vitro study demonstrated that rasagiline improves the expression of BDNF and other neurotrophic factors in neuroblastoma SH‐SY5Y cells.^132^ Furthermore, Sagi et al.^133^ revealed that rasagiline can activate TrkB and improve BDNF/TrkB signalling of nigrostriatal neurons in the post‐MPTP PD mouse model.

Safinamide is a reversible MAO‐B inhibitor, approved for the management of idiopathic PD in 2017. It also inhibits glutamate release and enhances the reuptake of dopamine and serotonin. Safinamide can block sodium and calcium channels with inhibition of opioid sigma receptors.^134^, ^135^ Different research stated that safinamide may be efficacious in the treatment of epilepsy and restless leg syndrome.^135^ Different studies highlighted the potential role of safinamide in the activation of BDNF/TrkB signalling.^135^, ^136^ Evidence from a preclinical study observed that safinamide has a neuroprotective effect against methamphetamine‐induced neurodegeneration by activating BDNF/TrkB signalling.^135^ Safinamide enhances the expression of BDNF and other neurotrophic factors in glial cell lines by reducing oxidative stress and mitochondrial dysfunction.^136^ Therefore, safinamide could be a promising drug for treating motor and non‐motor symptoms in PD patients.^137^ Moreover, safinamide attenuates neuroinflammation by inhibiting inflammatory signalling pathways like nuclear factor kappa B (NF‐κB) which is implicated in the dysregulation of BDNF/TrkB signalling.^138^

Ladostigil is an irreversible MAO‐B inhibitor with inhibition of acetylcholine‐esterase (AchE) used in the management of AD, PD, anxiety disorders and depression.^139^, ^140^ Ladostigil prevents the development of neurodegenerative diseases by enhancing BDNF/TrkB signalling via numerous molecular signalling.^139^, ^140^ Ladostigil has an antidepressant effect in rat model and attenuates MPTP‐induced nigrostriatal injury in mice by inhibiting AchE and improving BDNF/TrkB signalling.^141^ Ladostigil is more effective than other MAO‐B inhibitors in reducing PD neuropathology by increasing the expression of BDNF.^141^ It has been reported that ladostigil can reduce AD neuropathology by different mechanisms including antioxidant, anti‐inflammatory and anti‐apoptotic effects by enhancing BDNF expression.^142^

### 7.3 Dopamine receptor agonists

Dopamine receptor agonists are used in the management of PD, depression, hyperprolactinemia and restless leg syndrome.^143^ Two types of dopamine receptor agonists are found, ergot derivatives such as bromocriptine and pergolide, and non‐ergot derivatives such as pramipexole, ropinirole and rotigotine. Dopamine receptor agonists are mainly activating D2 receptor in the substantia nigra.^143^ In vitro study demonstrated that D2 agonist bromocriptine reduced the synthesis of BDNF at 24 h however; D1/D2 agonists such as pergolide and cabergoline increase the synthesis of BDNF at 6 h. D1 agonist SKF‐38393 increases the expression and synthesis of BDNF at 24 h.^144^ These findings indicated that non‐selective dopamine receptor agonists are more effective than selective D2 agonists in increasing BDNF expression. In vitro study showed that dopamine is necessary for the release of BDNF since dopamine antagonists abolish the release of BDNF.^145^ A case–control study on 48 PD patients and 48 healthy controls observed that pramipexole increased BDNF serum levels in PD individuals in comparison with the controls.^146^ Furthermore, the non‐ergot dopamine agonist rotigotine which activates all dopamine receptors is used in the management of PD.^147^ A recent experimental study conducted by Adachi et al.^148^ revealed that rotigotine promotes BDNF expression in the hippocampus and cerebral cortex in rats. In addition, rotigotine through activation of dopaminergic and 5HT receptors enhances the expression of antioxidant metallothionine in astrocytes.^149^ Metallothionine reduces brain neurodegeneration by enhancing the expression of BDNF.^150^

### 7.4 N‐methyl‐D‐aspartate receptor (NMDA) antagonists

NMDA antagonists are a class of medications that antagonize the effect of glutamate on the NMDA.^94^ NMDA antagonists such as amantadine and memantine are used in the management of PD and AD, respectively.^151^, ^152^ Amantadine is a non‐competitive NMDA antagonist that has marked antiviral effects and now is used in the management of PD, traumatic brain injury and dyskinesia.^153^ It has been shown that amantadine has antidepressant effects by improving BDNF expression.^154^ Notably, anaesthesia impairs memory and cognitive function by distorting synaptic plasticity. Amantadine has been shown to attenuate postoperative memory and cognitive dysfunction by inducing the expression of BDNF.^153^ In particular, a case series showed that treatment of depressed patients with 100‐200 mg/day amantadine reduced depressive symptoms within 1 week of treatment without recurrence of depressive symptoms following 2 weeks of discontinuation.^154^ The underlying antidepressant effect of amantadine may be related to the induction of BDNF expression.^154^ Rogoz et al.^155^ confirmed that co‐administration of amantadine and imipramine promotes BDNF gene expression in rat hippocampus. Furthermore, amantadine attenuates the dangerous consequences of acquired brain injury by promoting the expression of BDNF and other neurotrophic factors.^156^

On the other hand, memantine which is a non‐competitive NMDA antagonist attenuates glutamate excitotoxicity in AD. Memantine has a neuroprotective effect and can be used in the management of PD.^157^ It has been recently shown that memantine reduced motor deficit in a mouse model of neuromyelitis optica by improving BDNF signalling.^158^ Samartgis and colleagues revealed that memantine accelerates memory function by increasing BDNF expression.^159^ In particular, memantine reduces AD symptoms and pathology via increment of BDNF signalling in AD patients.^160^ Therefore, NMDA antagonists appear to have noteworthy neuroprotective effects against PD neuropathology by increasing the expression of BDNF.

### 7.5 TrkB agonists

TrkB agonists are effective against neurodegenerative diseases such as HD.^161^ TrkB signalling improves cell neuronal survival and synaptic plasticity. On the other hand, abnormal TrkB expression is associated with the onset and advancement of several neuropsychiatric and neurodegenerative conditions.^162^ TrkB is highly expressed in the hippocampus, cerebral cortex, cerebellum, retina, brain stem and spinal cord. As well, TrkB is observed in the peripheral nervous system.^161^, ^162^ TrkB is mainly activated by BDNF, neurotrophin 3 (NT3) and neurotrophin 4 (NT4).^162^ TrkB agonists may therefore be useful in the treatment of PD.

#### 7.5.1 Amitriptyline

Amitriptyline a tricyclic antidepressant used in the management of depression and chronic pain and has been shown to activate TrkB.^163^, ^164^ Amitriptyline activates both TrkA and TrkB leading to receptor dimerization and phosphorylation, and subsequent improvement of neurite outgrowth. A neuroprotective effect against kainic acid‐induced neuronal damage in mice is exhibited by Amitriptyline.^163^ The neuroprotective effect of amitriptyline is mediated by activation of both TrkA and TrkB, as TrkA antagonists abolish its neuroprotective effect.^163^ In vitro study demonstrated that amitriptyline has a neurotrophic effect on cultured primary cortical neurons through activation of TrkB and its downstream signalling.^165^ Moreover, amitriptyline via activation of TrkA and TrkB attenuates neuronal loss in the dorsal root ganglion and lidocaine‐induced neurodegeneration.^166^ It has been observed that amitriptyline attenuates the rotenone PD model through its anti‐inflammatory and antioxidant effects, and through the activation of TrkA and TrkB.^167^ A recent experimental study confirmed that amitriptyline ameliorates the integrity of the hippocampus and nigrostriatal pathway by reducing the deposition of α‐Syn and Lewy bodies in rat PD model.^168^ A previous randomized clinical study revealed that 3 months of treatment with amitriptyline 25 mg/day minimized depression and movement disorders in PD patients.^169^ Therefore, in virtue of its neuroprotective effect and activation of TrkB, amitriptyline has the potential to be efficacious in the treatment of PD and associated depression.

#### 7.5.2 Tropoflavin

Tropoflavin which is also known as 7,8 dihydroxyflavone (7,8‐DHF) is a natural flavone present in various herbs including *Tridax procumbens*, *Primula* and *Godmania aesculifolia*.^170^ Tropoflavin has poor oral bioavailability and can cross BBB, and a prodrug of tropoflavin known as R13 has higher efficacy and better pharmacokinetic profile, and is under progress for the treatment of AD.^171^ Tropoflavin is effective in the management of AD, PD, depression, brain ischemia schizophrenia and cognitive deficits. Tropoflavin is a potent and selective TrkB‐improving BDNF signalling.^172^ It has been reported that tropoflavin was effective against the experimental PD model and neurotoxicity. Structural analogues of tropoflavin such as diosmetin and norwogon also can activate TrkB.^172^ Tropoflavin improves synaptic plasticity and dopaminergic neurotransmission in the substantia nigra in the MPTP mouse model of PD.^173^ In addition, tropoflavin attenuates glutamate‐induced neurotoxicity by improving TrkB signalling.^172^

In addition, tropoflavin inhibits MPTP‐induced oxidative stress by inhibiting the expression of α‐Syn and by upregulating antioxidant enzymes.^173^ Tropoflavin is safe in PD patients so can be used with other anti‐PD agents.^171^ Till now, there are no clinical studies evaluating the neuroprotective of tropoflavin against the development of PD. These preclinical studies suggest that tropoflavin could be a promising therapeutic strategy for treating PD.

#### 7.5.3 Deoxygedunin

Deoxygedunin is a tetranortriterpenoid derived from the Indian neem tree. It is a selective agonist of TrkB and has a neuroprotective and neurotrophic effect.^174^ Deoxygedunin is rapidly absorbed after oral administration, rapidly acting within 2 h, has a long duration of action and can cross BBB.^174^ Has a weak affinity relative to tropoflavin, though it is more potent than tropoflavin. Deoxygedunin has a potent TrkB‐dependent antidepressant effect similar to that of tropoflavin in animal model studies.^175^ Different preclinical studies revealed that deoxygedunin protects the nigrostriatal pathway and dopaminergic neurons in the experimental PD model.^176^, ^177^ Deoxygedunin attenuates 6‐HAD and MPTP‐induced dopaminergic neurodegeneration in mice PD model.^176^ Deoxygedunin via activation of TrkB reduced neurotoxicity in experimental PD.^177^ Thus, deoxygedunin may be used as an adjuvant treatment in treating PD patients.

#### 7.5.4 N‐acetyl‐serotonin (NAS)

N‐acetyl‐serotonin also known as nor‐melatonin is an intermediate compound produced endogenously from serotonin and converted to melatonin. It activates melatonin receptors and TrkB.^178^ NAS is regarded as a neurotransmitter that has neuroprotective, antioxidant and anti‐inflammatory effects. Therefore, NAS may be effective in the management of neurodegenerative disorders including PD.^178^ NAS attenuates stress‐induced neurotoxicity through the activation of TrkB and inhibiting oxidative stress.^179^ It has been observed that NAS reduces the severity of experimental traumatic brain injury through the activation of TrkB and regulates autophagy function.^180^ Different studies highlighted that melatonin and serotonin are deregulated in PD patients.^181^, ^182^ Therefore, the administration of NAS may also regulate melatonin in PD patients.^183^ Thus, NAS through TrkB and regulation of melatonin may reduce PD neuropathology.

#### 7.5.5 COMT inhibitors

The catechol‐*O*‐methyltransferase (COMT) inhibitors such as entacapone and tolcapone are selective and reversible inhibitors of COMT enzyme, and are often used in the management of PD.^184^ Entacapone only inhibits the peripheral COMT enzyme though tolcapone can cross BBB and inhibit brain COMT enzyme. COMT inhibitors prolong the therapeutic effect of L‐dopa in patients with advanced PD.^184^ Findings from a preclinical study observed that entacapone improves hippocampal neurogenesis through activation of the BDNF–TrkB‐pCREB pathway in mice.^185^ In addition, entacapone enhances hippocampal synaptic plasticity by increasing the expression of hippocampal proteins dynamin I and synapsin I which control synaptic plasticity via BDNF–TrkB.^186^ Besides, tolcapone improves the activity of dopaminergic neurons in the cerebral cortex thereby augments cerebral neurological conductivity.^187^ Giakoumaki et al.^188^ revealed that tolcapone enhances working memory and psychomotor performance in animal model through modulation the BDNF expression. Thus, COMT inhibitors have neuroprotective effects against PD neuropathology by activating BDNF–TrkB signalling pathway.

#### 7.5.6 Anticholinergic drugs

The prescribing of anti‐muscarinic drugs in the management of PD has declined after the introduction of Ldopa and due to the risk of cognitive impairment. It has been documented that prolonged use of anti‐muscarinic drugs is associated with increasing risk of dementia.^189^ Biperiden is an anti‐muscarinic drug that treats movement disorders such as rigidity and to a lesser extent tremor in PD, but not for tardive dyskinesia. It was approved by the FDA in 1959 in treating PD, and it acts by antagonizing the Ach effect on the muscarinic receptors.^190^ Moreover, biperiden is effective in treating acute brain injury and post‐traumatic epilepsy through modulation of BDNF signalling.^191^ Zhou et al.^191^ found that biperiden has antidepressant‐like effects in mice by increasing the expression of BDNF signalling in the prefrontal cortex and hippocampus. A recent clinical trial showed that biperiden has an anti‐epileptogenic effect following traumatic brain injury through modulation of the expression of BDNF signalling.^192^

Furthermore, pridinol is a centrally acting muscle relaxant through anti‐muscarinic effect and modulation of brain polysynaptic reflexes. It was approved recently in 2021 in treating lumbago and torticollis.^193^ It has been shown that pridinol attenuates MPTP‐induced neurotoxicity by increasing BDNF signalling and modulation of NO signalling.^194^

Collectively, BDNF/TrkB activators promote neuroprotection and reduce PD neuropathology by enhancing dopaminergic neurotransmission in the substantia nigra. Hence, BDNF/TrkB signalling is reduced in PD and associated with disease severity and long‐term complications. Activation of BDNF/TrkB by specific activators may attenuate PD neuropathology. In this state, preclinical and clinical studies are recommended in this regards.

## 8 CONCLUSIONS

Parkinson's disease is a chronic, progressive neurodegenerative brain disease caused by the substantia nigra's dopaminergic neurons progressively degenerating. Dysfunction of BDNF is mainly implicated in PD neuropathology. Targeting of BDNF and TrkB by specific activators may attenuate PD neuropathology. Therefore, this review aimed to discuss the potential role of BDNF/TrkB activators in PD. BDNF promotes the survival of dopaminergic neurons and enhances functional activity of substantia nigra. BDNF has been shown to improve the release and uptake of dopamine. Deficiency of TrkB receptor triggers degeneration of dopaminergic neurons in the substantia nigra and accumulation of α‐Syn. BDNF plays a critical neuroprotective effect against the development of PD. Therefore, augmentation of BDNF signalling by activators could be a novel therapeutic strategy in the management of PD. In sum, BDNF/TrkB signalling is reduced in PD and associated with disease severity and long‐term complications. Activation of BDNF/TrkB by specific activators may attenuate PD neuropathology. In this state, preclinical and clinical studies are recommended in this regards.

## AUTHOR CONTRIBUTIONS

**Naif H. Ali:** Writing – review and editing (equal). **Hayder M. Al‐Kuraishy:** Resources (equal); software (equal); supervision (equal). **Ali I. Al‐Gareeb:** Conceptualization (equal); data curation (equal); visualization (equal). **Athanasios Alexiou:** Writing – original draft (equal). **Marios Papadakis:** Visualization (equal); writing – original draft (equal). **Ali Abdullah AlAseeri:** Writing – review and editing (equal). **Mubarak Alruwaili:** Writing – review and editing (equal). **Hebatallah M. Saad:** Validation (equal); writing – review and editing (equal). **Gaber El‐Saber Batiha:** Writing – review and editing (equal).

## FUNDING INFORMATION

This work was supported by the University of Witten‐Herdecke Germany.

## CONFLICT OF INTEREST STATEMENT

The authors declare no conflict of interest.

## Data Availability

Data sharing is not applicable to this article as no new data were created or analysed in this study.
